# Effect of ovarian steroids on vascular endothelial growth factor a expression in bovine uterine endothelial cells during adenomyosis

**DOI:** 10.1186/s12917-019-2222-0

**Published:** 2019-12-30

**Authors:** Martyna Lupicka, Anna Zadroga, Agata Szczepańska, Anna Justyna Korzekwa

**Affiliations:** Department of Biodiversity Protection, Institute of Animal Reproduction and Food Research of Polish Academy of Sciences, Tuwima 10 Str, 10-748 Olsztyn, Poland

**Keywords:** Adenomyosis, VEGFA, Ovarian steroids, Uterus, Bovine

## Abstract

**Background:**

Adenomyosis is a uterine dysfunction defined as the presence of endometrial glands within the myometrium. There is evidence that proangiogenic factors may play a role during the development of adenomyosis; however, exact mechanism remains unknown. The aim of the study was to determine the action of vascular endothelial growth factor A (VEGFA) in uterine tissue and uterine vascular endothelial cells during adenomyosis.

**Results:**

Uterine tissues were collected and examined for the presence and extent of adenomyosis. Gene and protein expression of VEGFA and its two receptors (VEGFR1 and VEGFR2) was evaluated with quantitative polymerase chain reaction and Western blotting, respectively, in endometrium and myometrium during adenomyosis. Immunolocalization of VEGFA and its receptors within uterine tissues during adenomyosis was also determined. In an in vitro experiment, endothelial cells from non-adenomyotic bovine uteri were treated with media conditioned by non-adenomyotic or adenomyotic uterine slices treated with 17-beta-oestradiol (E2) or progesterone (P4). Both gene and protein expression of VEGFR2 were elevated in endometrium in stages 3–4 of adenomyosis. Protein expression of VEGFA and VEGFR2 as well as VEGFA secretion were increased in endothelial cells treated with media conditioned by adenomyotic uterine slices after E2 treatment.

**Conclusions:**

Results suggest that VEGFA signalling is an important component, next to E2, that enhances VEGFA action and participates in adenomyosis development in cows.

## Background

Adenomyosis is a uterine dysfunction defined as the presence of endometrial glands within the myometrium [1]. The condition is observed in many female mammals, including cattle [2, 3]. As Ferreira et al. (2008) showed, in cows that failed to conceive after three or more insemination services (repeat breeder cows), uterine pathologies such as inflammation and degeneration of uterine morphology were predominant, followed by oviduct and ovarian pathologies [4]. Moreover, Campo et al. showed the decreased expression of genes responsible for successful embryo implantation in uterine tissue of women with adenomyosis [5]. In cows, adenomyotic changes in uterine tissue are divided into four stages [6]. Our previous studies showed that the frequency and progression of adenomyosis in cows are positively correlated with age [2], which is consistent with studies on women [7].

Adenomyosis is an oestrogen-dependent disease. In women with adenomyosis or endometriosis, the P450 aromatase is expressed in endometrial tissue, which is not observed in normal endometrium [8]. 17-beta-oestradiol (E2) promotes endometrial cell proliferation and new gland formation [9]. Our previous studies showed that oestradiol receptor α (ERα) immunoreactivity was higher in uterine tissue of cows with adenomyosis compared with cows without the condition [2]. Progesterone receptors (PR) are also expressed in endometrial cells infiltrating the myometrium but on the same level as in adjacent normal tissue [2].

According to the cyclic physiology of the endometrium, it undergoes changes that range from cellular proliferation, differentiation, apoptosis, and renewal [10]. Thus, angiogenesis plays an important role in tissue remodelling and is essential for pregnancy maintenance and proper uterine blood supply [11]. Uterine endothelial cells are sensitive for exogenous stimulation and can proliferate during reproductive cycles [12]. Ovarian hormones influence the growth and function of uterine vascular endothelial cells; however, it is not clear if the impact is direct, through steroid receptors, or indirect, through initiation of proangiogenic factors release. In general, it is suggested that E2 promotes angiogenesis and proliferation of endothelial cells and that progesterone (P4) may have the opposite influence [12–14]. However, these effects are not clearly understood and require further studies.

Among proangiogenic factors, vascular endothelial growth factor A (VEGFA) plays a key role in uterine vascularity [15, 16]. It acts on the endothelial cells of blood vessels through two membrane receptors: vascular endothelial growth factor receptor 1 (VEGFR1) and vascular endothelial growth factor receptor 2 (VEGFR2) [17]. Vascular endothelial growth factor A promotes the proliferation and migration of endothelial cells, as well as vascular permeability in both normal and pathologic conditions such as endometriosis [16, 18]. It is suggested that for this activity of VEGFA, the signalling through VEGFR2 is responsible, rather than through VEGFR1, which regulates normal vascularisation and is responsible for steady-state organisation of vessels [15]. Vascular endothelial growth factor A and its receptors are variably expressed in endometrial tissue during the cycle, which suggests that it depends on ovarian steroids [19, 20].

Angiogenesis in the uterus requires strict regulation; when the hormonal uterine environment is disturbed, pathological vascularisation occurs together with uterine fibroids, adenomyosis, and/or carcinomas [21]. In women, adenomyosis is accompanied by hyperplasia of uterine blood vessels, thus indicating the relation between this pathological condition and uterine vascularisation [22]. While endometrial cells infiltrate the myometrial layer during adenomyosis, new blood vessel formation is required [14]. It is suggested that adenomyotic epithelial cells, under the increased influence of E2, can undergo the transition into mesenchymal cells. In this process, the molecular signals are switched, and the cells begin to proliferate, invade the myometrial layer, and produce factors with proangiogenic properties, such as VEGFA [23]. Thus, in women with adenomyosis, E2 stimulates the expression of VEGFA in uterine glandular cells and in vascular endothelial cells as well, which results in increased angiogenesis [14, 24]. On the other hand, there is lack of studies concerning the action of P4 during adenomyosis. It is known that P4 may affect the proliferation of endothelial cells and VEGFA secretion. Monckedieck et al. reported progestins’ inhibitory effect on matrix metalloproteinases (MMP) and other angiogenic factors expression in endometrial cells [25]. However, this has not been extensively investigated against the background of uterine pathologies [14, 25].

There is a lack of information about pathological uterine angiogenesis in cows. However, hormonal disturbances during adenomyosis and immunological stress during endometritis may have a major influence on the formation of uterine blood vessels and proliferation of abnormal endometrial cells, which in turn may affect cow’s fertility [26–28]. The aim of the study was to determine and compare the action of VEGFA in uterine tissue and vascular endothelial cells in cows without and with adenomyosis. We hypothesised that during adenomyosis in the cow, 1) the expression and action of VEGFA is altered in the uterus and 2) ovarian steroids modulate the expression and action of VEGFA in uterine tissue. To test these hypotheses, we adopted the following specific objectives: in non-adenomyotic and adenomyotic cows, we compared 1) gene and protein expression of VEGFA and its receptors (VEGFR1 and VEGFR2 [VEGFA system]) in uterine tissue and 2) genes and proteins expression of the VEGFA system in cultured bovine vascular endothelial uterine cells (bVEUC) under the direct and indirect influence of ovarian steroids.

## Results

### Preliminary experiment. Classification of material according to adenomyosis extent

**Fig. 1:**
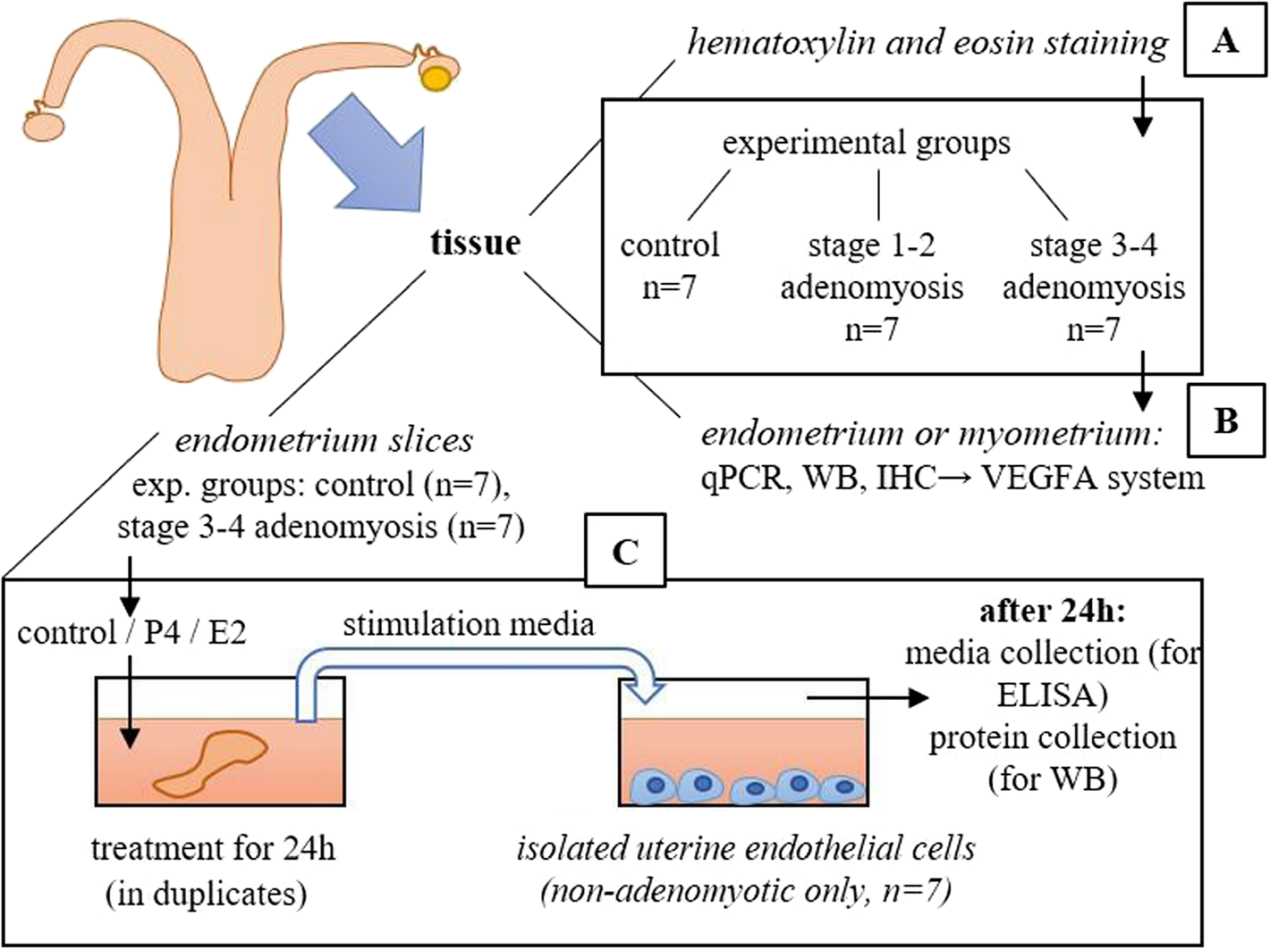
Experimental design of the study

**Fig. 2:**
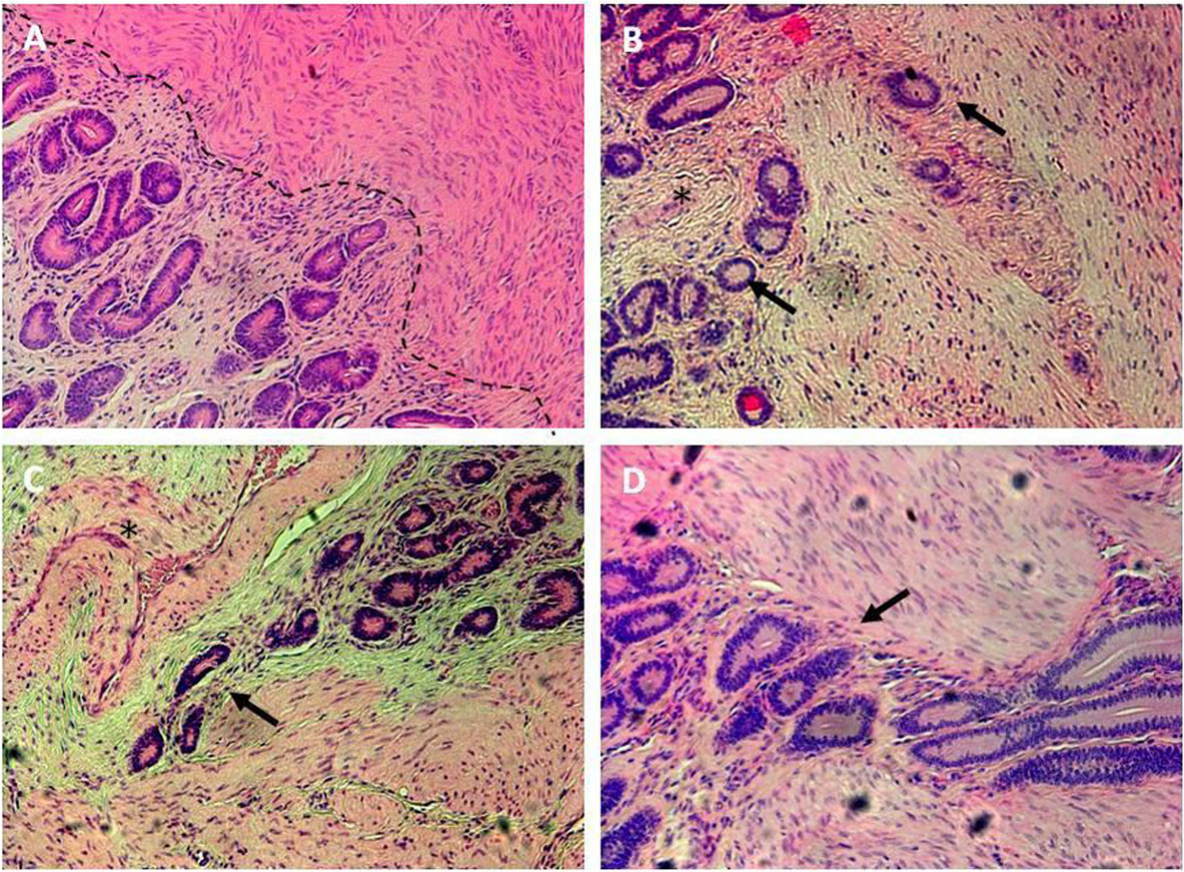
Representative pictures of hematoxylin/eosin stained bovine, uterine cross-sections of three experimental groups. **a** – control uterus without adenomyotic lesions, border between endometrium and myometrium was indicated with dashed line; **b** – adenomyotic uterus in stages 1–2; **c-d** – stages 3–4 of adenomyosis; arrows indicate uterine glands penetrating myometrium, asterisks indicate blood vessels. Magnification 100 x

Based on histological examination, tissues were classified into three categories: normal/control (without adenomyotic lesions), stage 1–2 adenomyosis, and stage 3–4 adenomyosis (Fig. 1a). In the first group of samples, the border between the endometrium and myometrium was clearly visible (Fig. 2a). In adenomyosis in stages 3–4, endometrial glands penetrated deeper within the myometrial layer (Fig. 2b-d). Blood vessels were observed in the myometrial layer of adenomyotic samples (Fig. 2b, c).

### Expression of VEGFA, VEGFR1, and VEGFR2 mRNA in uterine tissues during adenomyosis

The aim of the experiment was to determine if there were differences in VEGFA, VEGFR1, and VEGFR2 mRNA expression in uterine endometrium and myometrium tissues from different stages of adenomyosis (two experimental groups of stages 1–2 and 3–4) as compared with tissue with normal histology (control experimental group, Fig. 1b). Vascular endothelial growth factor A is crucial for angiogenesis regulation, and its action depends on the receptor which it binds to. Therefore, determination of the VEGFA system expression might provide an insight into the vascularisation state within uterine tissue during adenomyosis.

mRNA expression of VEGFA and its receptors was evaluated in three experimental categories in the separated endometrial and myometrial uterine tissues.

**Fig. 3:**
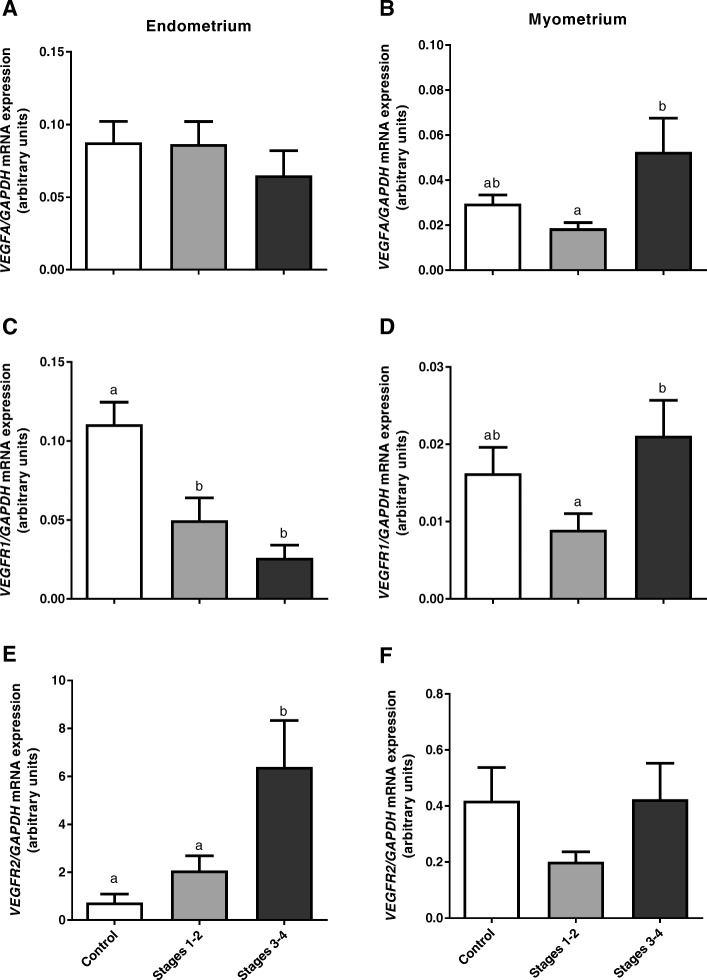
Expression of VEGFA (**a**, **b**), VEGFR1 (**c**, **d**) and VEGFR2 (**e**, **f**) mRNA in endometrial and myometrial bovine tissue during adenomyosis, determined by real-time PCR. Data were normalized against GAPDH. Bars represent the mean ± SEM. Different letters (**a**, **b**) indicate statistically significant differences between healthy tissues (Control, *n* = 7) and tissues from stages 1–2 (*n* = 7), and stages 3–4 (*n* = 7) of adenomyosis extent. *P* < 0.05 was considered as significant, and was determined using one-way ANOVA, followed by Bonferroni’s post-hoc test

Vascular endothelial growth factor A gene expression was not significantly different in the endometrium during adenomyosis (*P* > 0.05, Fig. 3a). However, it was increased in myometrial samples with adenomyosis in stages 3–4 when compared with stage 1–2 adenomyosis (*P* < 0.05, Fig. 3b).

Vascular endothelial growth factor receptor 1 mRNA expression was decreased during adenomyosis in endometrial tissue (*P* < 0.05, Fig. 3c). In the myometrium, the expression pattern for the VEGFR1 gene was similar to that of VEGFA, and it was increased in stage 3–4 adenomyosis as compared with stage 1–2 (*P* < 0.05, Fig. 3d), although this difference was not significant when comparing both stages to control tissues (*P* > 0.05, Fig. 3d).

Vascular endothelial growth factor receptor 2 mRNA expression was higher in the endometrium during stages 3–4 adenomyosis as compared with both control and stages 1–2 adenomyosis (P < 0.05, Fig. 3e). However, in the myometrium, no significant changes were observed in VEGFR2 mRNA expression (P > 0.05, Fig. 3f).

### Immunolocalization and expression of VEGFA, VEGFR1, and VEGFR2 protein in uterine tissues during adenomyosis

The aim of the experiment was to determine if there were differences in VEGFA, VEGFR1, and VEGFR2 protein expression in uterine endometrium and myometrium tissues from different stages of adenomyosis (two experimental groups of stages1–2 and 3–4) as compared with tissues with normal histology (control experimental group). In addition, the aim was to determine where VEGFA system proteins are localised specifically within the uterine tissue in the control group and in the adenomyotic groups as well (Fig. 1b).

**Fig. 4:**
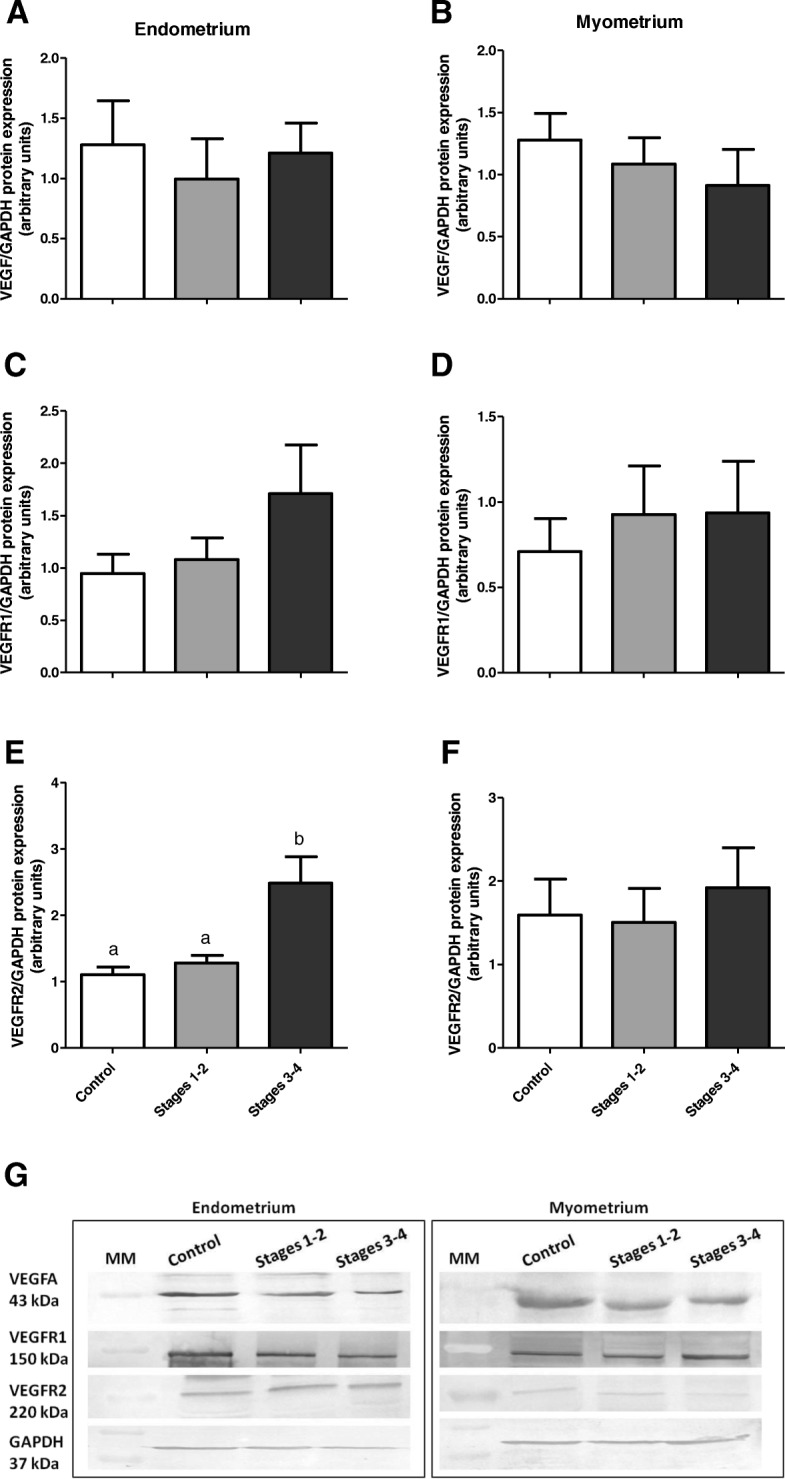
Protein expression of VEGFA (**a**, **b**), VEGFR1 (**c**, **d**) and VEGFR2 (**e**, **f**) in endometrial and myometrial bovine tissue during adenomyosis, determined by Western blotting. Data were normalized against GAPDH. Bars represent the mean ± SEM. Different letters (**a**, **b**) indicate statistically significant differences between healthy tissues (Control, *n* = 7) and tissues from stages 1–2 (*n* = 7), and stages 3–4 of adenomyosis extent (*n* = 7). *P* < 0.05 was considered as significant, and was determined using one-way ANOVA, followed by Bonferroni’s post-hoc test. Representative blots for VEGFA, VGFR1, VEGFR2 and GAPDH are shown below the graphs (**g**). MM: molecular weight marker

Vascular endothelial growth factor A and VEGFR1 protein expression did not differ significantly between experimental groups in either endometrial or myometrial tissue (*P* > 0.05, Fig. 4a-d).

Vascular endothelial growth factor receptor 2 protein expression was increased in endometrial tissue from uteri with stages 3–4 adenomyosis when compared with the control endometria and those obtained from uteri with stages 1–2 adenomyosis (*P* < 0.05, Fig. 4e). In myometrial tissues, there were no significant differences in VEGFR2 protein abundance between experimental groups (P > 0.05, Fig. 4f).

**Fig. 5:**
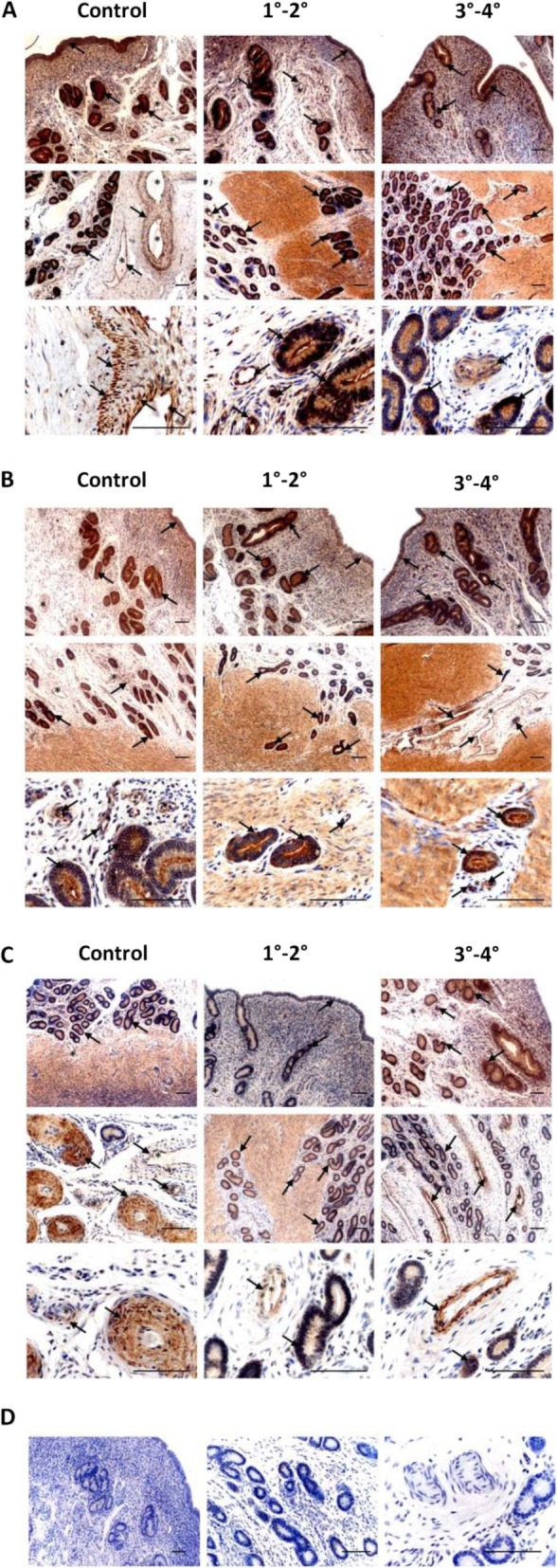
Immunodetection of VEGFA (**a**), VEGFR1 (**b**) and VEGFR2 (**c**) in uterine tissues of cows without adenomyosis (Control, *n* = 7) and with different degrees of the dysfunction (1°-2° and 3°-4°, *n* = 7 per each group). Arrows indicate the most intense immunoreaction; asterisks indicate blood vessels; negative non-specific IgG isotype controls (D). Scale bars: 100 μm

Vascular endothelial growth factor A and its receptors were localised in uterine tissues in all experimental groups. Both VEGFA and VEGFR1 were abundantly localised in uterine luminal, glandular epithelial, and endometrial endothelial cells of vessels in control slides and slides of different adenomyosis stages (Table 1). VEGFA and VEGFR1 were also abundantly localised within glandular cells and vessels within adenomyotic lesions (Fig. 5a and b, Table 1). VEGFR2 was also localised in glandular and endothelial cells within all uterine cross sections; however, in slides from uterine tissues with stages 3–4 adenomyosis, the immunoreaction in glandular epithelium and vascular endothelium appeared to be stronger than for control slides and tissues from stages 1–2 adenomyosis (Fig. 5c, Table 1).

**Table 1:** Relative abundance of VEGFA, VEGFR1 and VEGFR2 in: non-adenomyotic uterine tissue (control), tissue at the stages 1–2 of adenomyosis (1^o^-2^o^) and tissue at the stages 3–4 of adenomyosis (3^o^ -4^o^). Abundance level was determined by IHC staining intensities indicated as follows: + weak staining, ++ moderate staining, +++ strong staining, N/A not applicable

	VEGFA			VEGFR1			VEGFR2		
	control	1^o^-2^o^	3^o^-4^o^	control	1^o^-2^o^	3^o^-4^o^	control	1^o^-2^o^	3^o^-4^o^
endometrial glands	+++	+++	+++	++	++	++	+	+	+
endometrial blood vessels	++	++	++	++	++	++	++	++	++
endometrial stroma	+	+	+	+	+	+	+	+	+
myometrium	++	++	++	++	+	++	+	+	+
glands within adenomyotic lesions	N/A	+++	+++	N/A	++	++	N/A	+	++
myometrial blood vessels	++	++	++	++	+	+	++	++	++

*VEGFA* vascular endotelial growth factor A, *VEGFR1* vascular endotelial growth factor receptor 1, *VEGFR2* vascular endotelial growth factor receptor 2, *control* tissue without adenomyosis, *1*^*o*^*-2*^*o*^ adenomyosis stages 1–2, *3*^*o*^*-4*^*o*^ adenomyosis stages 3–4

### Expression of VEGFA and its receptor proteins in bVEUC treated with stimulation media conditioned by healthy or adenomyotic uterine slices incubated with or without P4 and E2

The aim of the experiment was to compare changes in VEGFA system protein expression and VEGFA secretion by bVEUC, derived from normal uteri, under the influence of potentially angiogenic factors released by P4- and E2-treated endometrial slices of healthy (*n* = 7) and adenomyotic cows (stages 3–4, *n* = 7, Fig. 1c). In the experiment, the effect of P4 and E2 is expected as rather indirect on bVEUC VEGFA production, since P4 and E2 are probably mostly utilized by the tissue slices. There is still a possibility of some amount of these hormones present in stimulation media after transfer to bVEUC which would be then the direct effect on these cells.

**Fig. 6:**
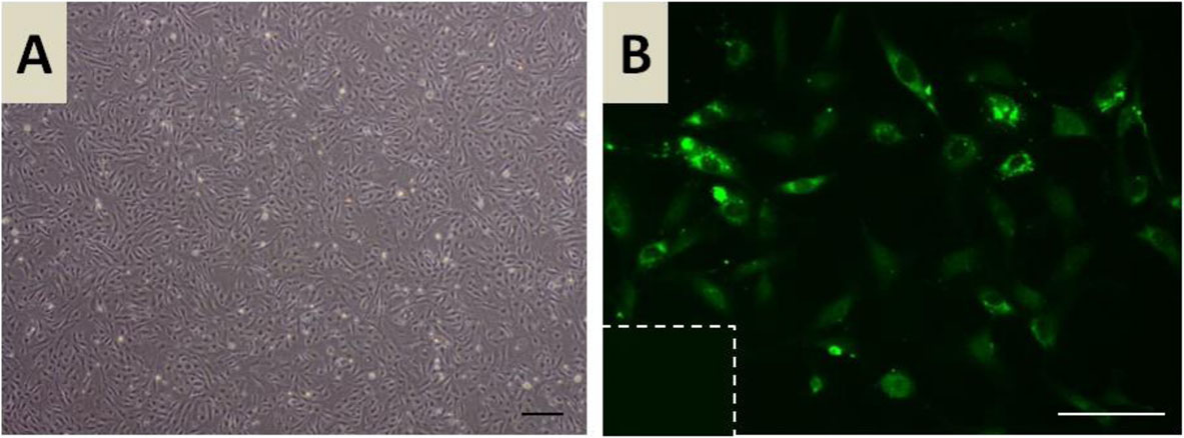
Representative picture of in vitro cultured endothelial cells isolated from bovine uterine tissue (**a**). Representative picture of immunofluorescence staining (green) for Von Willebrand factor of uterine endothelial in vitro cultured cells (**b**). Scale bars: 100 μm

The homogeneity of bVEUC isolated and cultured in this experiment was confirmed with observed rhomboidal, typical for endothelium, morphology of cells (Fig. 6a). Moreover, immunofluorescence staining for the endothelial cells marker, Von Willebrand factor, also confirmed the presence of that marker in obtained and cultured cells (Fig. 6b).

**Fig. 7:**
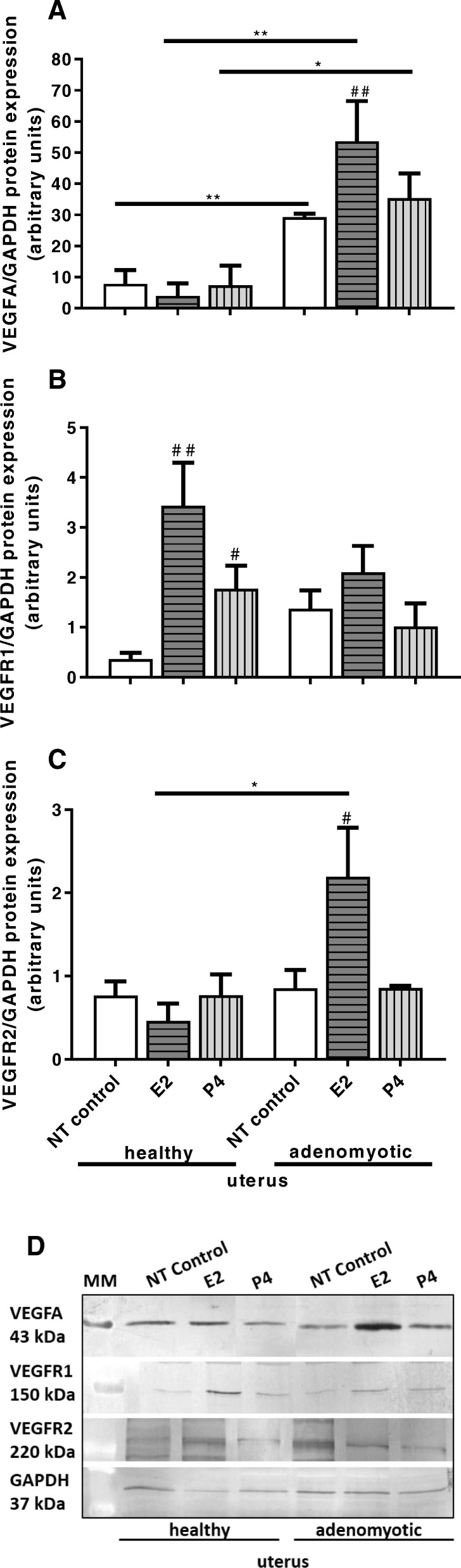
Protein expression of VEGFA (**a**), VEGFR1 (**b**) and VEGFR2 (**c**), measured with Western blotting, in uterine endothelial cells cultured in vitro and stimulated for 24 h with media conditioned by uterine slices from non-adenomyotic uterine tissue (healthy, *n* = 7) and from adenomyosis in stages 3–4 (adenomyotic). Additionally, uterine slices were treated with E2 or P4 and non-treated control (NT control). Data were normalized against GAPDH. Bars represent the mean ± SEM. Asterisks indicate statistically significant differences in effects of same treatments between non-adenomyotic and adenomyotic group. * *P* < 0.05, ** *P* < 0.01. Pound signs indicate statistically significant differences between treatments comparing to non-treated control within groups of non-adenomyotic or adenomyotic uterine tissues. ## *P* < 0.01, # *P* < 0.05. *P* < 0.05 was considered as significant, and was determined using two-way ANOVA, followed by Bonferroni’s post-hoc test. Representative blots for VEGFA, VGFR1, VEGFR2 and GAPDH are shown below the graphs (**d**). MM: molecular weight marker

Vascular endothelial growth factor A protein expression was increased in endothelial cells treated with media conditioned by adenomyotic uterine slices as compared with cells treated with media from control uteri, which indicated increased basal expression of VEGFA due to adenomyosis (Fig. 7a, *P* < 0.01). Similarly, VEGFA abundance was increased in bVEUC treated with media from adenomyotic endometrial slices incubated with E2 and P4 as compared with cells treated with media from non-adenomyotic slices also treated with hormones, respectively E2 and P4 (Fig. 7a, *P* < 0.01 and *P* < 0.05, respectively). However, the P4 effect on bVEUC was not different when comparing that treatment to its NT control, which is cells treated with stimulation media from adenomyotic slices but without any hormones. Whereas, E2 induced an increase in VEGFA abundance in cells treated with media from adenomyotic uterine slices incubated with E2 when compared with cells incubated with media from nontreated adenomyotic uterine slices (Fig. 7a, *P* < 0.01).

Vascular endothelial growth factor receptor 1 protein abundance was increased in bVEUC treated with media conditioned by healthy uterine slices incubated with E2 and P4 when compared with cells incubated with nontreated healthy uterine slices (Fig. 7b, *P* < 0.05).

Vascular endothelial growth factor receptor 2 protein expression was increased in bVEUC treated with media obtained after incubation of adenomyotic uterine slices treated by E2, when compared with the respective cells treated with control media from healthy uterine slices incubated with E2 (Fig. 7c, *P* < 0.05). In addition, E2 induced an increase in VEGFR2 abundance in cells treated with media from adenomyotic uterine slices incubated with E2 when compared with cells incubated with media from nontreated uterine slices (Fig. 7c, *P* < 0.05).

**Fig. 8:**
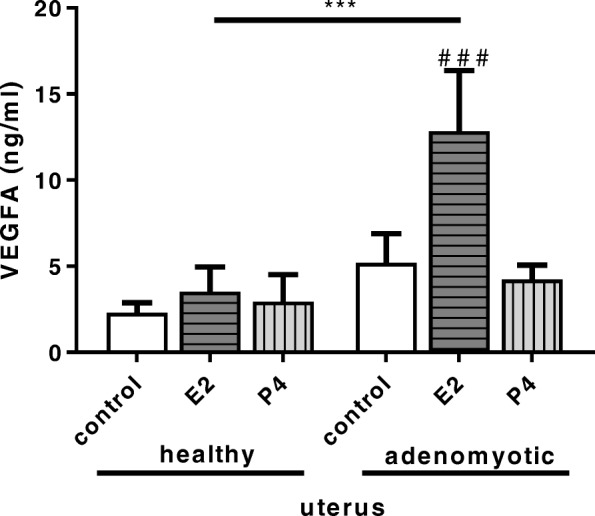
Secretion of VEGFA, measured with ELISA, by uterine endothelial cells cultured in vitro and stimulated for 24 h with media conditioned by uterine slices from non-adenomyotic uterine tissue (healthy) and from adenomyosis in stages 3–4 (adenomyotic). Additionally, uterine slices were treated with E2 or P4 and non-treated control (control). Bars represent the mean ± SEM. *** *P* < 0.001 indicates statistically significant difference in effects of E2 treatment between non-adenomyotic and adenomyotic group. Pound signs indicate statistically significant differences between treatments comparing to non-treated control within groups of non-adenomyotic or adenomyotic uterine tissues. ### *P* < 0.001. *P* < 0.05 was considered as significant, and was determined using two-way ANOVA, followed by Bonferroni’s post-hoc test

The concentration of VEGFA secreted into medium was increased when bVEUC were treated with media conditioned by adenomyotic uterine slices incubated with E2 as compared with cells treated with media from healthy uterine slices also incubated with E2 (Fig. 8, *P* < 0.001) and compared with cells treated with media from adenomyotic endometrial slices without additional hormonal factors (Fig. 8, *P* < 0.001).

## Discussion

In this study, we showed for the first time the relationship between VEGFA and its receptors’ expression and the indirect effect of ovarian steroids on the expression during adenomyosis in the cow. We compared mRNA and protein expression of VEGFA, VEGFR1, and VEGFR2 in two main compartments of uterine tissue: endometrium and myometrium at different stages of adenomyosis, which demonstrated indeed change in the abundance of these genes and proteins between experimental groups. In particular, the increase in VEGFR2 expression was observed in the endometrium during stages 3–4 of adenomyosis. We also created an in vitro experimental model for evaluation of the indirect, through stimulated endometrial slices, effect of P4 and E2 on VEGFA system expression in uterine endothelial cells during adenomyosis. For this purpose, uterine tissue slices with or without adenomyosis were stimulated with steroids, and in the next step, the medium after culture with secretory products of uterine cells was transferred directly to bVEUC. Such model allowed us to observe how changes caused by steroids during adenomyosis influence the VEGFA system expression of uterine endothelial cells. We observed the elevation in VEGFA expression and secretion after E2 stimulation. That result indicates that adenomyotic vascularisation process, induced by VEGFA, is oestrogen dependent in the cow.

Adenomyosis causes an increase in uterine angiogenesis, which was previously described by Hickey et al. (1999) and Benagiano and Brosens (2012) in women but not yet explored in ruminants [21, 29]. Moreover, the genetic polymorphism of VEGFA and fibroblast growth factor (FGF), major factors related to the formation of new blood vessels, is associated with the risk of adenomyosis in women, which indicates that pathological angiogenesis may contribute to the development and progression of adenomyosis [30, 31]. In our study, the increase in both mRNA and protein expression of VEGFR2 in the endometrium during adenomyosis in stages 3–4 was observed when compared with that of normal uterus and stages 1–2 adenomyosis. However, this was not the case in the myometrium. This result suggests that the angiogenic and mitogenic signal, stimulating the formation of adenomyotic lesions in cow, arises from the endometrium. Another study showed that in the endometrium of women with adenomyosis there is no change in vasculature when compared to normal endometrium. However, the authors also found that the density of microvessels was significantly increased in adenomyotic lesions in myometrium [32]. These data in compilation with our findings, suggest that angiogenic signals in adenomyosis may arise from endometrium and affect the areas where adenomyotic foci form. Excessive stimulation through VEGFR2 in the endometrium may induce the invasive potential of endothelial cells in that compartment, therefore supporting formation of adenomyotic nests penetrating myometrium [32].

In the bovine uterus, VEGFA stimulates the proliferation and migration of endothelial and smooth muscle cells, thereby enabling the cyclic development of new blood vessels and stimulating the permeability changes in the blood vessels [17, 19]. In the cow during oestrus, the mRNA expression of VEGFA was higher than in the luteal phase, which indicates E2 engagement in the expression regulation, whereas the protein concentration was the highest in the early luteal stage [19]. Protein expression of VEGFR1 was on the same level during the oestrous cycle, which suggests that VEGFA functions mediated by this receptor play a constant role in bovine endometrium during the reproductive cycle. On the other hand, VEGFR2 protein expression was the highest in mid- and late- luteal stages of the oestrous cycle, thus, during that time angiogenic activity of VEGFA through VEGFR2 may be the most pronounced [19]. Interestingly, in our in vitro experiment, we showed that potentially mitogenic and angiogenic factors released by normal uterine tissue slices, also from these treated with E2 or P4, did not cause an increase in VEGFA protein abundance nor its secretion, which suggests it was a balanced environment for VEGFA function. However, the dysfunctional condition, adenomyosis, caused a response in increased VEGFA production under the influence of E2. These results suggest that E2 is a powerful modulator of mitogenic and angiogenic activity of endothelial cells in bovine uterus, not in a direct way but through indirect communication between endometrial cells, depending on their state, and vascular endothelial cells. VEGFA can also act in a paracrine and autocrine manner on stromal and epithelial uterine cells, promoting their proliferation. As reported in literature, increased VEGFA expression in adenomyotic tissue may contribute to abnormal endometrial cell proliferation and glandular infiltration within the myometrium [33]. Therefore, based on the observed increased production of VEGFA by bVEUC exposed to the stimulation media from adenomyotic tissue explants treated with E2, we conclude that during adenomyosis endothelial cells also participate in accelerating lesions development.

It is suggested that while VEGFR1 binds VEGFA, its bioavailability for VEGFR2 is limited, which constitutes a preventive mechanism from the excessive neovascularisation, since VEGFR2 is the receptor responsible for proangiogenic signalling of VEGFA [15]. In our in vitro experiment, we showed that VEGFR1 might be the guardian of regulation of VEGFA actions in the environment of increased E2 stimulation in a healthy tissue, since VEGFR1 expression was increased in endothelial cells incubated with control explants (non-adenomyotic) treated with both hormones, E2 and P4, while during adenomyosis this silencing mechanism was not observed, and VEGFR2 protein abundance was higher under E2 influence instead.

In most studies, human and mice uterine endothelial cells expressed oestradiol receptor β (ERβ) but not ERα. Both receptors, however, can equally activate oestrogen target genes expression [34–37]. Studies show that E2, through its receptors, either on uterine cells or uterine endothelium has rather promoting effect on angiogenesis, causing increased VEGFA and VEGFR2 expression [37, 38]. There is less studies concerning the localisation and expression of oestrogen receptors in endothelial cells in ruminant uterus [2]. Oestradiol receptor α and ERβ mRNA and protein are shown to be expressed in ovine uterine aortic endothelium [39]. On the other hand, PR expression in uterine endothelial cells in any species is questionable. On the gene level, PR were detectable in human endometrial endothelial cells; however, the protein level was very low and below the detection limits of the immunohistochemistry method [34]. Nevertheless, ovarian steroids may affect the uterine endothelial cells indirectly, by complex networks with other uterine cells [38]. Endometrial tissue consists of cells that express steroid hormone receptors [40, 41], and in response to the stimulation, these cells can produce many factors modulating angiogenesis (e.g., VEGFA, FGF, MMPs) [42, 43]. Moreira et al. (2011) were the first to indicate the increased expression of MMP-2, a protein that modulates angiogenesis, in glandular nests penetrating the myometrial layer, and in uterine muscle cells adjacent to infiltrating endometrium in cows [26]. In the present study, there was a substantial increase in VEGFR1 protein expression after both E2 and P4 treatment, but simultaneously, no changes were observed in VEGFA and VEGFR2 protein expression in bVEUC stimulated with medium collected from healthy bovine uterine slices. In bVEUC stimulated with medium obtained from adenomyotic cows, the elevation in VEGFA, regardless the hormonal treatment, and VEGFR2 protein expression, ender E2 influence, was demonstrated. No differences in VEGFA were observed in these cells after stimulation with P4 when comparing to its adenomyotic NT control group (no hormonal treatment, stimulated with media from adenomyotic tissue). The lack of significant difference within adenomyotic group for P4 treatment, suggests that P4 itself did not augment angiogenic activity. Therefore, based on that result we conclude that the stimulation media from adenomyotic tissue explants generally increased the basal VEGFA production, despite hormonal treatment, and only E2 further multiplied that effect because that treatment significantly increased VEGFA even comparing to adenomyotic NT control. These results together indicate that during adenomyosis, E2 may indirectly affect the VEGFA signalling pathway of binding VEGFA by VEGFR2 in endothelial endometrial cells in the cow. During the physiological conditions, the VEGFA pathway system is also modified by steroids, but in this case, it relates to binding VEGFA by VEGFR1. According to these findings P4 is not significantly involved in cell signalling with engagement of VEGFA during adenomyosis.

## Conclusions

In the summary, we showed that VEGFR2 receptor expression is increased in adenomyotic endometrium, indicating a potentially excessive mitogenic and angiogenic stimulation coming from VEGFA, even though the factor itself was not elevated. Moreover, E2 significantly affected both VEGFA and VEGFR2 protein abundance and VEGFA secretion by bovine vascular endothelial cells in an adenomyotic environment, which suggests that the dysfunction of signalling between E2, endometrial cells, and vascular endothelial cells occurs during the disease. The results of the present study suggest that VEGFA signalling is another important component, next to E2, that affects the development of adenomyosis in cows.

## Methods

### Material collection

Uterine tissues were obtained *post-mortem* at the meat-processing plant “Warmia” (Biskupiec, Poland) from 21 Holstein/Polish Black and White cows (75%/25%, respectively). The age of used animals ranged between 5 to 7 years old. The material was transported on ice to the laboratory. The stage of the oestrous cycle (day 8–12) was evaluated by macroscopic examination of the ovaries, corpus luteum and uterus [44] and further confirmed by P4 determination in peripheral blood plasma using radioimmunoassay. Before slaughter, animals were examined by per rectum ultrasound examination and age record was obtained. Blood samples were collected from the jugular vein. Animals were culled from the herd for economic reasons and herd renewal.

Further, in the laboratory, endometrial and myometrial tissue fragments were dissected from uterine wall, the middle segment of its horn ipsilateral to the corpus luteum. Tissue pieces were then divided into three portions: one for histo- and immunohistochemical staining, one frozen and stored in − 86 °C for further mRNA and protein expression determination, and one used for immediate isolation and culture of uterine endothelial cells.

### Histochemical staining and preliminary division of the material

Uterine tissue fixed in 4% paraformaldehyde (PFA) was processed for haematoxylin and eosin staining according to a standard protocol. Cross sections were used for adenomyosis classification as described previously [3]. Briefly, uterine samples were classified as normal/control (*n* = 7) if endometrial-myometrial border was clearly distinguishable and no endometrial glands were present within myometrial layer. If uterine glands were slightly crossing the mucosal-myometrial border, tissues were classified as adenomyosis at stage 1 and 2 (*n* = 7). If glandular nests were identified deep within myometrium, reaching serosa layer, samples were classified as stages 3 and 4 of adenomyosis (*n* = 7). Division of material was performed based on previously established classification by Katkiewicz et al. (2005) [6].

### Incubation of uterine slices

Based on haematoxylin and eosin staining, uterine tissue fragments were used to retrospectively determine the control group (*n* = 7) and adenomyotic group (*n* = 7, only stage 3 and 4). Tissue fragments containing only endometrium were cut with scissors and weighed (approximately 30 mg per slice). Slices were rinsed thoroughly in phosphate-buffered saline (PBS), placed into separate wells of a 24-well plate containing 1 ml of Dulbecco’s Modified Eagle’s medium (DMEM) with 0.1% bovine serum albumin (BSA), preincubated for 2 h, then incubated for 24 h with or without ovarian steroids (P4, dose: 10^− 6^ M and E2, dose: 10^− 7^ M) in a tissue incubator with shaking at 37.5 °C, in a humidified atmosphere of 5% CO2 and 95% air. Per each treatment group: control without ovarian steroids (NT control), P4 or E2, two slices of endometrial tissue per animal were used in separate wells (performed in technical duplicates). Doses of steroids were based on preliminary studies and previous reports in the literature [45, 46]. After stimulation, media were collected and stored at − 20 °C until isolated bVEUC reached confluence.

### Isolation of vascular endothelial uterine cells and treatment with stimulation media

Uterine horns obtained from individuals retrospectively classified as healthy based on haematoxylin and eosin staining (*n* = 7) were cut to expose the inner wall of the uterus. Endometrial and myometrial layers containing blood vessels were dissected with scissors. The tissue fragments were minced until homogenous material was obtained. Diced uterine tissue was dispersed in 50 ml of M199 medium containing 1% BSA, 20 μg/ml gentamicin, 2 mg/ml collagenase I, 1 mg/ml deoxyribonuclease, and 0.1 g dispase. The enzyme solution with the tissue was incubated for 30 min in 37.5 °C with stirring. After digestion, the cell suspension was filtered through a filter mesh to remove undigested tissue fragments. Cells were washed by spinning down two times (10 min at 300 x g, at 4 °C), suspended in PBS with 1% BSA, and after final wash mixed with magnetic tosylactivated beads coated with BS-1 (the vascular endothelial cell-specific lectin; Thermo Fisher Scientific, Dynabeads™ M-280 Tosylactivated, 14,203, Wilmington, DE) at a bead:endothelial cell ratio of 1:3. Endothelial cells conjugated with the beads were washed with PBS and sorted out using a magnet [47] (according to the supplier’s protocol). Obtained uterine vascular endothelial cells were resuspended in Endothelial Cell Basal Medium (Sigma Aldrich, 210–500) with 10% foetal calf serum and antibiotics (gentamicin/amphotericin B). Cells viability was determined by staining with 0.3% trypan blue. Endothelial cells were seeded at a density of 1 × 10^6^ of living cells/ml into 24-well culture plates (Thermo Fisher Scientific; A1142802) and cultured at 37.5 °C in a humidified atmosphere of 5% CO2, 95% air. The medium was changed every 2 days until the confluence was reached (approximately 3–4 days). The cell culture homogeneity was confirmed by Von Willebrand factor fluorescent immunostaining of cells in culture.

In the next step, bVEUC were washed with fresh medium and incubated for 24 h with 1 ml per well of medium conditioned by endometrial slices, obtained from healthy (*n* = 7) and adenomyotic (*n* = 7, stages 3–4) individuals (non-treated or treated with E2 or P4, two wells per treatment, technical duplicates). After incubation cells were harvested in lysis buffer and frozen at − 86 °C for further Western blotting analysis. Culture media were collected and frozen at − 20 °C for determination of VEGFA concentration.

### Total RNA isolation

For transcript level analysis, total RNA was extracted from homogenized tissues using trizol (Sigma-Aldrich, T9424) according to standard RNA extraction protocol. The concentration and purity of obtained RNA were assessed with a NanoDrop 1000 instrument (Thermo Fisher Scientific, ND-1000). We evaluated RNA Integrity Number (RIN) for a representative group of samples (from each experimental group) on Bioanalyzer 2100 (Agilent). Samples were characterized by RIN between 8 and 10. Samples were stored at − 80 °C until reverse transcription. One microgram of each sample of total RNA was reverse-transcribed into cDNA in a total volume of 20 μl using the High Capacity cDNA Reverse Transcription Kit (Applied Biosystems, 4,368,813, Cheshire, UK) according to the supplier’s protocol. Complementary, single stranded, DNA was stored at − 20 °C until used for real-time polymerase chain reaction (PCR).

### Real-time PCR

The primer sequences for determining the mRNA relative abundance of VEGFA, VEGFR1, and VEGFR2 genes were designed using Primer Express Software 3 (Applied Biosystems). The housekeeping gene, glyceraldehyde-3-phosphate dehydrogenase (GAPDH), was chosen based on previous results that confirmed its stable expression in uterine tissue [48], and two other reference genes: beta actin (ACTB) and 18S ribosomal RNA (RN18S). The primer sequences, GenBank accession numbers, genes and expected product size are detailed in Table 2. Quantitative real-time PCR method was used for determination of the relative abundance of VEGFA, VEGFR1, and VEGFR2 genes in the uterine tissues at the mRNA level with the Applied Biosystems 7900 system (Applied Biosystems, Foster City, CA, USA). The reaction mixture (10 μL) contained 7 μL of SensiFAST SYBR Hi-ROX Master Mix (Bioline Reagents, BIO-92002, London, UK; 3 mM MgCl2, 1x SYBR green, and 0.5 μM forward and reverse primers) and 10 ng of reverse-transcribed cDNA (3 μL). A standard curve was plotted based on serial dilutions of the appropriate cDNA to evaluate amplification efficiency. Amplification was performed according to real-time PCR reaction program described previously [3]. After amplification, melting curves were plotted by stepwise increases in temperature (60 to 95 °C) to ensure that a single product was amplified and that no primer-dimer structures were formed. All samples were run in duplicates. Dissociation curve analysis was carried out after each real-time PCR assay to confirm that only one amplification product was present. Data were calculated using the ΔΔCt method. The stability of the reference genes was determined using the NormFinder program. mRNA relative abundance data are expressed relative to the housekeeping gene GAPDH and are presented as arbitrary units. Control reactions without the template or primers were ran to confirm that the products were free from primer-dimers and genomic DNA contamination, respectively.

**Table 2:** Oligonucleotide sequences used for real-time PCR

Gene	Oligonucleotide sequences	Product size (bp)	GeneBank
*VEGFA*	FWD 5′- CCATGAACTTTCTGCTCTCTTGG −3′	148	NM_174216.2
	REV 5′- CTGCGCTGGTAGACATCCAT − 3′		
*VEGFR1*	FWD 5′- ACGGTAGCCATCAGCAGTTC − 3′	188	NM_001191132.2
	REV 5′- GCTTTGCAGTGATACACGCC −3’		
*VEGFR2*	FWD 5′- CGCTCAACAGGATGGCAAAG −3’	85	NM_001110000.1
	REV 5′- AGGCAGAGAGAGTCCCGAAT −3’		
*ACTB*	FWD 5′-CCAAGGCCAACCGTGAGAAAAT-3’	256	K00622
	REV 5′- CCACATTCCGTGAGGATCTTCA-3’		
*RN18S*	FWD 5′-AAGTCTTTGGGTTCCGGG-3’	365	AF176811
	REV 5′-GGACATCTAAGGGCATCACA −3’		
*GAPDH*	FWD 5′-CACCCTCAAGATTGTCAGCA-3’	103	BC102589
	REV 5′-GGTCATAAGTCCCTCCACGA-3’		

*VEGFA* vascular endotelial growth factor A, *VEGFR1* vascular endotelial growth factor receptor 1, *VEGFR2* vascular endotelial growth factor receptor 2, *ACTB* beta actin, *RN18S* 18S ribosomal RNA, *GAPDH* glyceraldehyde-3-phosphate dehydrogenase

### Western blotting

Proteins were extracted from cultured cells by incubation with lysis buffer containing 50 mM Tris-HCl (pH 8.0), 150 mM NaCl, 5 mM EDTA, 0.1% sodium dodecyl sulfate (SDS), 1% Triton X-100, 0.5% sodium deoxycholate, and protease inhibitors (Sigma, P8340). Protein concentrations were assessed spectrophotometrically by the Bradford method and the lysates were stored at − 86 °C until further analysis. Samples containing 30 μg of protein were dissolved in SDS gel-loading buffer, heated to 95 °C for 4 min, and separated by 10% SDS–polyacrylamide gel electrophoresis (PAGE) for VEGFA and GAPDH and 8% SDS-PAGE for VEGFR1 and R2. Separated proteins were transferred into PVDF membrane in transfer buffer. After transfer, non-specific protein binding was blocked by incubation of membranes with 5% non-fat dry milk prepared in TBS-T buffer for 1.5 h at room temperature (RT), the membranes were incubated overnight with a 1 μg/ml of an anti-VEGFA antibody (Abcam, ab46154), 10 μg/ml of an anti-VEGFR1 and R2 antibodies (Abcam, ab32152 and ab45010, respectively), and 1 μg/ml of an anti-GAPDH antibody (Sigma, G8795) as a reference. Membranes were incubated with a 1:20,000 dilution of secondary polyclonal anti-rabbit alkaline phosphatase-conjugated antibodies (Sigma, A3687) for 1.5 h at RT. According to the datasheet from the manufacturer’s protocol, the band 43 kDa was identified for VEGFA, 150 kDa for VEGFR1, 220 kDa for VEGFR2, and 37 kDa was identified for GAPDH. Proteins were visualised by incubating the membranes with 0.4 mg/ml nitro-blue tetrazolium chloride and 0.2 mg/ml 5-bromo-4-chloro-3-indolylphosphate toluidine salt (Sigma, 72,091) suspended in Tris-buffered saline (pH 9.5). The intensity of the Western Blot bands was analysed with the The Quantity One 1-D Analysis Software (Bio-Rad, Hercules, CA, USA).

### Vascular endothelial growth factor a concentration determination

Measurements of VEGFA concentration in culture media were performed using commercially available enzyme immunoassay kit (Kingfisher Biotech, INC, VS0286B-002). Each sample was run in technical duplicate. The standard curve for VEGFA ranged from 0.23–15 ng/ml, and the effective dose for 50% inhibition (ED 50) of the assay was 0.55 ng/ml. The intra- and interassay coefficients of variation were 4.1 and 12.2%, respectively.

### Immunohistochemistry and immunofluorescence

Tissue samples fixed in 4% PFA were embedded in paraffin and sliced on microtome to obtain 7-μm sections and subjected to immunostaining. Each tissue slide was deparaffinized by incubation in 37 °C for 15 min and washing with xylene, followed by rehydration by washing with solutions of decreasing ethanol concentration. Heat induced epitope retrieval in citrate buffer was also performed. Endogenous peroxidase was blocked by treating the tissue slices with hydrogen peroxide in methanol and washed in 0.1 M PBS. Subsequently, blocking with 10% normal goat serum (Sigma, G9023) for 1 h at room temperature (approx. 23 °C; RT) was performed to block unspecific binding of antibodies. Tissue sections were then incubated overnight at 4 °C with 1:100 dilution of an anti-VEGFA (Abcam, ab46154), anti-VEGFR1, and anti-VEGFR2 antibodies (Abcam, ab32152 and ab45010); washed in PBS, and incubated for 1 h with 1:25,000 dilution of biotinylated anti-rabbit (Vectastain ABC Kit; Vector Laboratories, PK 4001, Burlingame, CA, USA) antibodies. The excess of antibodies was washed off, sections were incubated for 45 min with the ABD reagent in PBS and washed again. Vascular endothelial growth factor A, VEGFR1 and VEGFR2 were visualised by incubation for 2–3 min in solution containing 0.3 mg/ml 3,3′-diaminobenzidine (DAB) in 0.01% hydrogen peroxide. Sections were dehydrated and mounted with DPX (Park Scientific Ltd., D-11601, Northampton, UK). Simultaneously, isotype and no primary antibody controls were performed. Observations and photographs were made with a light microscope (Nikon FXA).

To confirm the homogeneity of bovine uterine endothelial cell cultures, the detection of the endothelial cell marker, Von Willebrand factor, was performed. Briefly, a portion of cells was cultured in a chamber slide, and cells were first washed with PBS to remove culture medium leftovers and fixed with 2% PFA. Then, the cells were washed three more times with PBS and blocked for 1 h with 10% normal donkey serum. Cells were then incubated for another 2 h at RT with anti–Von Willebrand factor antibodies (Abcam, ab6994) in a dilution of 1:200. After the incubation, cells were washed three times with PBS and then incubated for 50 min with secondary antibodies conjugated with DyLight 488 fluorophore (anti-rabbit, AbD Serotec, STAR36D488GA) in a dilution of 1:1000. Cells were washed three times with PBS and covered with a coverslip in mounting medium. Nonspecific IgG antibodies were used as a negative control. Cells were observed under the fluorescence microscope (Zeiss, Axiovision software).

Staining intensity was assessed by two independent, manual measurements, for tissue slides from 4 different animals per each experimental group, using following scoring system: + weak staining, ++ moderate staining, +++ strong staining [49].

### Statistical analyses

Statistical analysis of the results was performed using GraphPad Prism (GraphPad PRISM, Version 6.0, San Diego, CA, USA).

The relationship between the mRNA relative abundance and protein expression of VEGFA, VEGFR1 and R2 were determined using one-way ANOVA followed by a Bonferroni post hoc test. Analysis concerned comparison between healthy and two adenomyotic groups: 1–2 stages and 3–4 stages.

A two-way ANOVA followed by the Bonferroni test was performed for analyses of VEGFA, VEGFR1 and R2 and VEGF protein expression and VEGFA concentration in bVEUC stimulated with P4 and E2 received from healthy and adenomyotic uterine slices.

All numerical data are expressed as the arithmetic mean ± SEM. Differences at *P* < 0.05 were considered as statistically significant.

## Data Availability

The datasets used and/or analysed during the current study are available from the corresponding author on reasonable request.

## Abbreviations

ACTB: Beta actin
BSA: Bovine serum albumin
bVEUC: Bovine vascular endothelial uterine cells
DAB: 3,3′-diaminobenzidine
DMEM: Dulbecco’s Modified Eagle’s medium
E2: Oestradiol
ED 50: Effective dose for 50% inhibition
ERα: Oestradiaol receptor α
ERβ: Oestradiol receptor β
FGF: Fibroblast growth factor
GAPDH: Glyceraldehyde-3-phosphate dehydrogenase
MMP: Matrix metalloproteinases
PAGE: Polyacrylamide gel electrophoresis
PBS: Phosphate-buffered saline
PCR: Polymerase chain reaction
PFA: Paraformaldehyde
PR: Progesterone receptors
RIN: RNA Integrity Number
RN18S: 18S ribosomal RNA
RT: Room temperature
VEGFA: Vascular endothelial growth factor A
VEGFR1: Vascular endothelial growth factor receptor 1
VEGFR2: Vascular endothelial growth factor receptor 2
